# Internalized stigma and caregiver burden among caregivers of adolescents with non-suicidal self-injury: a moderated mediation analysis

**DOI:** 10.3389/fpsyt.2025.1640466

**Published:** 2025-09-26

**Authors:** Yuan Qin, Ying Yu, Jiao Liu, Jiming Duan, Huixia Liao, Bo Yang

**Affiliations:** ^1^ Department of Psychiatry, Chongqing Mental Health Center, Chongqing, China; ^2^ School of Nursing, Zunyi Medical University, Zunyi, China; ^3^ Department of Nursing, Chengdu Fifth People’s Hospital, Chengdu, China; ^4^ Sleep Medical Center, Chongqing Mental Health Center, Chongqing, China; ^5^ Department of Nursing, Chongqing Mental Health Center, Chongqing, China

**Keywords:** non-suicidal self-injury, caregiver burden, internalized stigma, moderated mediation model, caregiver

## Abstract

**Background:**

Adolescent non-suicidal self-injury (NSSI) constitutes a critical global health challenge, generating profound internalized stigma and familial burden among caregivers. However, the underlying mechanisms governing the relationship between these factors remain poorly understood.

**Methods:**

A cross-sectional survey was conducted. A sample of 385 caregivers of adolescents with NSSI completed the Internalized Stigma of Mental Illness Scale, Generalized Anxiety Disorder 7-Item Scale, Social Support Rating Scale, and Family Burden Scale of Disease. Moderated mediation analysis was employed to examine the dual mechanisms whereby anxiety mediates and social support moderates the stigma-to-burden pathway among caregivers.

**Results:**

The results revealed a pronounced direct effect of internalized stigma on caregiver burden (*β* = 0.265, *p* < 0.001) after adjusting for gender and age. Anxiety demonstrated partial mediation in this relationship (*β* = 0.254, *p* < 0.001), and social support significantly moderated the anxiety-mediated pathway (*β* = -0.007, *p* < 0.01), confirming convergent psychosocial pathways linking stigma to caregiver burden.

**Conclusions:**

This study confirms a psychosocial pathway where internalized stigma exacerbates caregiver burden through anxiety-mediated mechanisms, while social support exerts moderated effects by mitigating anxiety’s mediating potency. These findings highlight the need for comprehensive strategies to reduce stigma, lower anxiety, and strengthen social support to break the cycle of ongoing stress for caregivers.

## 1 Introduction

Non-suicidal self-injury (NSSI) is clinically conceptualized as the deliberate, self-directed infliction of tissue damage in the absence of suicidal intent (1). Significantly, globally pooled lifetime prevalence among adolescents reaches 22.0% (2), with Chinese adolescents demonstrating a notably higher prevalence of 24.7% (3). Crucially, beyond its direct harm to adolescents (4), NSSI imposes multidimensional familial burdens (5), particularly on caregivers who experience profound stress, social isolation, and internalized stigma (6). While caregiver burden, conceptualized as the objective and subjective strain from caregiving, affects 25.2% of primary caregivers in mental health contexts (7), critical knowledge gaps persist regarding how stigma internalization mechanistically translates into burden in NSSI-specific caregiving. Existing models, including the stress-process framework, recognize stigma as a chronic stressor but fail to account for the bidirectional stigma-burden dynamic unique to NSSI caregivers, where perceived societal blame exacerbates self-stigmatization (8). Although studies report correlations between internalized stigma and burden, they predominantly neglect moderating buffers like anxiety and social support, which may explain outcome heterogeneity (9). Therefore, further investigation into the mechanisms through which internalized stigma influences caregiver burden is warranted to develop targeted interventions that address both the adolescent’s needs and the psychological well-being of their caregivers.

Several studies have demonstrated that caregivers who internalize stigma related to mental illness tend to report higher levels of caregiving burden (10, 11). For instance, a recent study found that stronger illness-related stigma was disproportionately represented in caregivers with moderate and high burden profiles (12). Similarly, research on family functioning and NSSI has shown that caregivers’ expressed emotions, including criticism and invalidation, are associated with increased NSSI behaviors in adolescents, which in turn contribute to greater caregiving challenges (13). These findings suggest that internalized stigma may not only reflect caregivers’ negative attitudes toward the adolescent’s behavior but also contribute to their emotional and psychological distress, thereby increasing the burden they bear (14). Moreover, when caregivers internalize stigma related to NSSI, they may perceive the adolescent’s behavior as a reflection of personal failure or moral weakness, which can lead to feelings of guilt, shame, and helplessness. These negative emotions can further intensify the caregiving burden, as caregivers may struggle to provide effective support while also grappling with their own emotional challenges (15). Additionally, the interpersonal model of NSSI suggests that family dynamics, including parental criticism and invalidation, play a crucial role in the onset and maintenance of NSSI (16). When caregivers internalize stigma, they may inadvertently reinforce these negative family dynamics, creating a cycle of emotional distress and caregiving burden. Thus, we hypothesized that internalized stigma among caregivers of NSSI adolescents may demonstrate a significant positive association with caregiver burden (Hypothesis 1).

Research on families of individuals with mental illness has demonstrated that internalized stigma is associated with increased caregiver burden, partly due to the emotional toll it takes on the caregivers themselves (17). This emotional toll often manifests as anxiety, which can further exacerbate the challenges of caregiving (18). A previous study revealed that 35.3% of caregivers for adolescents with NSSI experienced clinically significant anxiety symptoms (19). Moreover, studies have shown that individuals who internalize stigma tend to experience higher levels of anxiety and depression, which in turn affect their ability to cope with caregiving responsibilities (20). In the context of adolescents with NSSI, caregivers may internalize stigma related to NSSI, leading to negative self-perceptions and emotional distress (21). These distressing emotions can then contribute to anxiety, which may further intensify the caregiving burden by impairing the caregiver’s ability to provide effective support and manage daily challenges (15). Furthermore, the stress-coping model of mental illness stigma suggests that emotional responses, such as anxiety, play a critical role in how individuals process and respond to stigma. When caregivers internalize stigma, they may engage in maladaptive coping strategies, such as rumination or avoidance, which can perpetuate anxiety and increase the likelihood of experiencing high levels of burden (22). Therefore, we posited that anxiety serves as a mediator in the stigma-to-burden pathway (Hypothesis 2).

Prolonged exposure to social stigma in high-stress environments may lead caregivers with mental disorders to internalize societal negative perceptions, developing self-stigmatizing beliefs such as “I don’t deserve assistance” (10). These beliefs frequently result in a rejecting attitude toward patients, but caregivers still had difficulty withdrawing their care when the patients were already in distress (23). Social support functions as a crucial protective buffer, mitigating the adverse impacts of stigmatization on mental health outcomes (24). Research on individuals with mental illness has found that higher levels of social support are associated with lower levels of anxiety and depression, and can reduce the impact of internalized stigma on mental health (25). Empirical studies demonstrate that perceived social support buffers caregiving burden-induced psychological distress, while independently predicting positive mental health outcomes (26). In Chinese samples, familial cohesion and extended kinship networks attenuate stigma-induced anxiety by reframing mental illness as a shared challenge rather than an individual failing, thereby enhancing coping efficacy (27). Extensive empirical evidence confirms the significant moderating role of social support across diverse caregiver populations, demonstrating cross-cultural consistency in its buffering effects against psychological distress (28–30). Thus, we hypothesized that social support may moderate the first-stage mediation pathway of internalized stigma and anxiety (Hypothesis 3).

Collectively, this study employs a moderated mediation framework to examine three core components: (1) the direct association between internalized stigma and caregiver burden (H1); (2) the mediating role of anxiety in this pathway (H2); and (3) the buffering effect of social support moderating the stigma-to-anxiety relationship (H3). By integrating mediation and moderation within a unified model, this research resolves prior theoretical oversimplifications of the stigma-burden dynamic, advancing a contextually nuanced framework specific to NSSI caregivers’ lived realities.

## 2 Methods

### 2.1 Design and participants

A convenience sampling methodology was implemented to enroll 385 clinically primary caregivers of adolescents with NSSI, all receiving inpatient psychiatric care at a tertiary mental health institution in Chengdu, Sichuan Province, China. Following rigorous application of established data quality criteria, 374 eligible datasets were included in the final analysis, corresponding to a 97.1% valid participation rate. In this study, the inclusion criteria were: (1) adolescents aged 13–18 years meeting DSM-5 criteria for NSSI, as confirmed by clinicians; (2) primary caregivers, legal guardians, or direct financial supporters of the patients. Exclusion criteria: (1) adolescents with significant somatic comorbidities (to avoid confounding effects on self-injury evaluation); (2) caregivers with severe health impairments (potentially affecting caregiving ability or introducing bias); (3) individuals undergoing major trauma unrelated to the adolescent’s condition (to minimize external stressor interference).

### 2.2 Measures

#### 2.2.1 Internalized Stigma

Developed by Ritsher et al. (31), the 29-item Internalized Stigma of Mental Illness Scale (ISMI) quantifies stigma internalization via a psychometrically robust multidimensional structure encompassing five clinically validated subdomains: alienation (perceived social marginalization), stereotype endorsement (internalization of prejudicial beliefs), perceived discrimination (subjective experiences of bias), social withdrawal (behavioral avoidance patterns), and stigma resistance (active coping mechanisms). Utilizing a 4-point Likert system (anchored at 1 = strongly disagree to 4 = strongly agree), cumulative scores demonstrate positive linear correlation with the severity of internalized stigma pathology. This instrument has established robust psychometric validity within culturally adapted Chinese cohorts (Cronbach’s α = 0.87) (32). The measure demonstrated robust internal consistency reliability in our psychometric evaluation (Cronbach’s α = 0.913).

#### 2.2.2 Anxiety

The Generalized Anxiety Disorder 7-item scale (GAD-7), pioneered by Spitzer (33), quantitatively measures DSM-5-aligned anxiety symptomatology severity using a retrospective self-report methodology with a 14-day recall period. This measurement is operationalized through seven diagnostic criteria measured on a 4-point anchored response continuum (0 = none to 3 = persistent daily manifestation), generating summative scores (theoretical range 0-21) with validated interpretive thresholds: minimal (0-4), mild (5-9), moderate (10-14), and severe (15-21). The Chinese version has demonstrated strong psychometric properties (Cronbach’s α= 0.89) in clinical samples (34). In our study, it exhibited excellent internal consistency (Cronbach’s α= 0.905).

#### 2.2.3 Social support

Developed by Xiao Shuiyuan (35), the Social Support Rating Scale (SSRS) quantifies perceived social support across three dimensions (encompassing affective connectedness, tangible assistance accessibility, and support mobilization capacity), is operationalized through 10 psychometrically calibrated items. Scoring architecture follows a validated algorithm, producing summative metrics with a theoretical range of 12-66. The Chinese adaptation has established cultural validation through population-specific standardization studies demonstrating satisfactory reliability indices (Cronbach’s α = 0.78-0.85) (36). Within our study sample, the Chinese version exhibited high reliability (Cronbach’s α = 0.740).

#### 2.2.4 Caregiver burden

The Family Burden Scale of Disease (FBS) (37), conceptualized through rigorous psychometric development to assess multifaceted caregiver burden across physical, psychological, social, and economic domains, operationalizes 24 psychometrically calibrated items structured across six constructs: financial strain, daily functioning impairment, leisure activity restriction, familial conflict, health deterioration, and psychological distress. Employing a 3-point Likert response framework (0 = never to 2 = always/severely), the cumulative scoring directly reflects burden severity. Previous validation studies in Chinese psychiatric populations reported strong psychometric properties (Cronbach’s α= 0.89-0.92) (38). In our cohort, the Chinese adaptation demonstrated exceptional reliability (Cronbach’s α = 0.953).

### 2.3 Procedures

The survey was administered through Questionnaire Star, a widely utilized online data collection platform in China, using well-validated self-report measures. Trained researchers provided participants with standardized explanations regarding the study’s objectives, procedures, and completion guidelines. Following informed consent, electronic questionnaire access codes were distributed. To enhance data reliability, researchers conducted on-site supervision to address participant queries during survey completion. To ensure participant anonymity, all collected data were fully de-identified at the point of entry. Questionnaire Star’s platform automatically anonymized responses by replacing personal information with unique numerical IDs, and no IP addresses or device identifiers were stored. Confidentiality was maintained by restricting data access exclusively to the research team via password-protected servers. During on-site supervision, researchers emphasized voluntary participation and the right to withdraw without penalty. All procedures adhered to the Declaration of Helsinki and were approved by the Ethics Committee of the Fourth People’s Hospital of Chengdu (Approval No [2022]71), which included explicit review of confidentiality protocols.

### 2.4 Data analysis

Data were analyzed using SPSS 26.0. Prior to conducting statistical analyses, the distribution of the data was assessed to ensure the assumptions of the statistical methods were met. Descriptive statistics, including mean, standard deviation, skewness, and kurtosis, were calculated to evaluate the data distribution and identify potential deviations from normality. Normality assumptions were assessed using the Shapiro-Wilk test. Additionally, multicollinearity among continuous variables was evaluated using the variance inflation factor (VIF), with a threshold of 5.0 considered acceptable. To address potential common method bias, Harman’s single-factor test was conducted (39). Subsequent analyses included descriptive statistics, Pearson correlations, and regression analyses to explore relationships among variables. The Hayes’ PROCESS macro for SPSS was employed to test the hypothesized mediation and moderated mediation models (Model 4 and Model 7). Bias-corrected 95% confidence intervals (CIs) were generated using 5,000 bootstrap iterations with a percentile approach. Statistical significance was determined when the 95% CIs did not include the null parameter value.

## 3 Results

### 3.1 Assessment of multicollinearity

To evaluate potential multicollinearity among predictors, the Variance Inflation Factor (VIF) analysis was conducted. All computed VIF values fell within acceptable limits, well below the conservative threshold of 5(the maximum value being 3.312), confirming the independence of predictor variables.

### 3.2 Common method deviation test

Results indicated no single factor explained >40% variance (15.95% variance explained), suggesting common method bias was unlikely to influence our findings substantially.

### 3.3 Correlation analysis

**Table 1** details descriptive statistics and Pearson correlation coefficients for internalized stigma, anxiety, social support, and caregiver burden. Correlation analysis revealed significant positive correlations between caregivers’ internalized stigma and anxiety (r = 0.516, *p* < 0.01), as well as caregiver burden (r = 0.569, *p* < 0.01). Conversely, stigma exhibited an inverse association with social support (r = -0.530, *p* < 0.01). Anxiety demonstrated negative linkages to social support (r = -0.488, *p* < 0.01) but positive covariation with caregiver burden (r = 0.714, *p* < 0.01). Social support was inversely related to caregiver burden (r = -0.563, *p* < 0.01).

**Table 1:** Demographic and clinical characteristics with correlation analysis (N = 374).

Variables	*M* ± SD	1	2	3	4	5	6
1. Internalized stigma	55.99 ± 10.86	1					
2. Anxiety	7.42 ± 4.34	0.516**	1				
3. Social support	37.59 ± 8.35	-.530**	-0.488**	1			
4. Caregiver burden	24.82 ± 11.34	0.569**	0.714**	-0.563**	1		
5. Gender		0.206**	0.304**	-0.190**	0.346**	1	
6. Age	43.70 ± 5.60	0.107*	0.025	-0.06	0.02	-0.173**	1

*p < 0.05.**p < 0.01.*** *p* < 0.001.

### 3.4 Testing for mediation

Mediation analysis was performed using the PROCESS macro (v3.3) in SPSS 26.0 with Model 4 configuration. Parameters included 5,000 bootstrap resamples at 95% bias-corrected confidence intervals, covaried for gender and age. All variables were standardized prior to analysis following mediation testing protocols. **Tables 2**, **3** summarize the complete dataset.

**Table 2:** Mediating role of anxiety in caregiver burden pathways.

Model		Fit index			Test outcome	
Dependent variable	Predictors	*R*	*R2*	*F*	*β*	*t*
Anxiety		0.554	0.307	54.606***		
	Gender				0.433	4.624***
	Age				0.002	0.243
	Internalized Stigma				0.472	10.547***
Caregiver Burden		0.761	0.579	126.798***		
	Gender				0.265	3.529***
	Age				0.000	0.020
	Internalized Stigma				0.265	6.652***
	Anxiety				0.538	13.263***

***p < 0.001.

**Table 3:** Estimated effects in mediation analysis.

Effects	*β*	Std. error	95% boot. CI LL	95% boot. CI UL
Indirect effects	0.254	0.032	0.193	0.319
Direct effects	0.265	0.040	0.187	0.343
Total effects	0.519	0.042	0.435	0.602

As presented in **Tables 2** and **3**, caregivers’ internalized stigma significantly predicted anxiety severity (*β* = 0.472, *t* = 10.547, *p* < 0.001) and demonstrated independent effects on caregiver burden (*β* = 0.265, *t* = 6.652, *p* < 0.001). Anxiety strongly predicted caregiver burden (*β* = 0.538, *t* = 13.263, *p* < 0.001), with mediation analysis confirming anxiety’s partial mediating role between internalized stigma and caregiver burden (indirect effect = 0.254, 95%CI [0.193,0.319]; total effect = 0.519, 95%CI [0.435,0.602]). Direct effects remained significant (effect = 0.265, 95%CI [0.187,0.343]), with all confidence intervals excluding zero. Anxiety mediated 48.94% of total impacts.

### 3.5 Testing for moderated mediation

**Figure 1:**
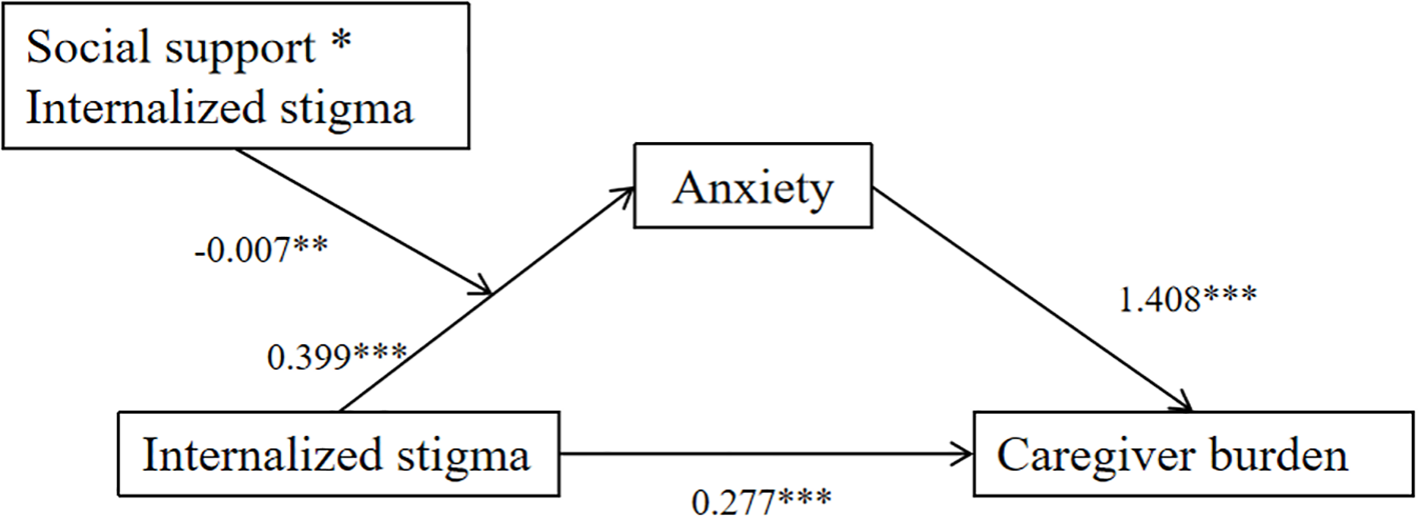
Moderated mediation: social support in the internalized stigma anxiety pathway. *p < 0.05, **p < 0.01, *** p < 0.001.

Moderated mediation analyses were conducted using the PROCESS macro Model 7 (SPSS v26.0) to examine social support’s moderated effects on the anxiety-mediated stigma-burden pathway. Full analytical outputs appear in **Table 4**, which details the conditional process effects of stigma internalization and anxiety. The result demonstrates that internalized stigma exerted a significant adverse effect on anxiety (*β* = 0.399, *t* = 4.806, *p* < 0.001), while anxiety served as a potent negative predictor of caregiver burden (*β* = 1.408, *t* = 13.263, *p* < 0.001). Social support demonstrated an inverse association with anxiety (*β* = -0.252, *t* = -2.060, *p* < 0.05). Critically, the interaction between social support and internalized stigma significantly predicted anxiety levels (*β* = -0.007, *t* = -3.313, *p* < 0.01), with this moderating effect visualized in **Figure 1**.

**Table 4:** Social support as a moderator of internalized stigma-anxiety mediation.

Model		Model evaluation metrics			Significance	
Dependent variable	Predictors	*R*	*R2*	*F*	*β*	*t*
Caregiver burden		0.761	0.579	126.798***		
	Internalized stigma				0.277	6.652***
	Anxiety				1.408	13.263***
	Gender				3.011	3.529***
	Age				0.001	0.019
Anxiety		0.617	0.381	45.206***		
	Internalized stigma				0.399	4.806***
	Social support				-0.252	-2.060*
	Social support * Internalized stigma				-0.007	-3.313**
	Gender				1.672	4.317***
	Age				-0.001	-0.033

*p < 0.05. **p < 0.01. ***p < 0.001.

Social support levels were parametrically demarcated using ±1SD thresholds: high (*M* + 1*SD*) and low (*M*-1*SD*) subgroups based on mean score deviations. **Table 5** presents stratified mediation effects across social support levels. Results indicated that under low social support conditions (*M-1SD*), internalized stigma exerted a significant adverse impact on anxiety (effect = 0.273, 95% CI = [0.192, 0.354]. Conversely, when social support was high (*M+1SD*), stigma internalization remained a significant predictor of anxiety, though with reduced effect magnitude (effect = 0.108, 95% CI = [0.016, 0.191].

**Table 5:** Differential indirect effects by social support tertiles.

Social support	*β*	Std. error	95% boot. CI LL	95% boot. CI UL
Low social support	0.273	0.041	0.192	0.354
High social support	0.108	0.045	0.016	0.191

**Figure 2:**
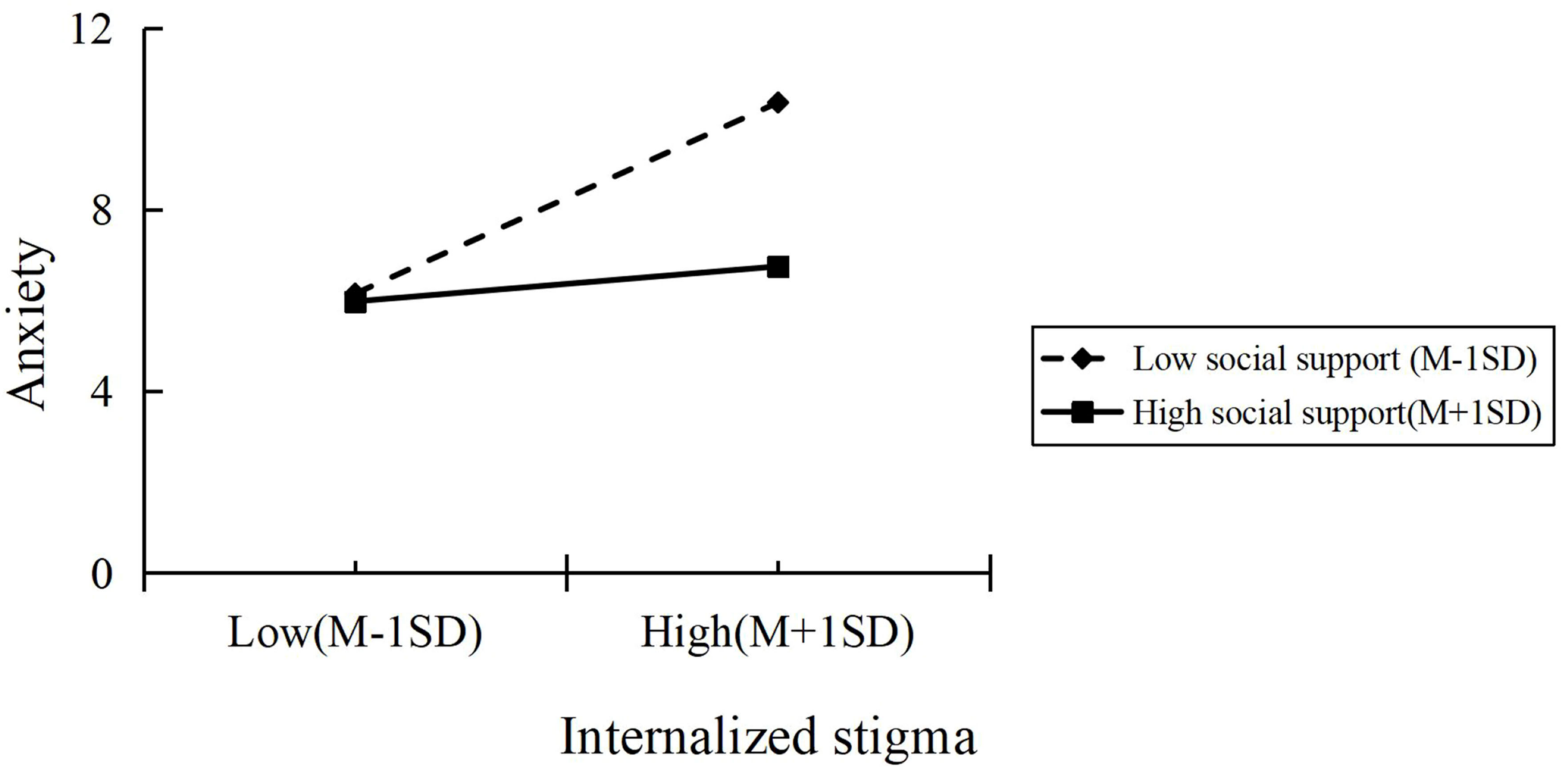
Simple slopes.

Simple slopes analysis was implemented to clarify social support’s regulatory effects within the stigma-anxiety pathway, with conditional effects at ±1SD thresholds as visualized in **Figure 2**. The plotted trajectories revealed differential patterns across support levels: lower social support was associated with steeper anxiety escalation per unit stigma increase, whereas higher support buffers exhibited attenuated anxiety progression. This demonstrates anxiety-mediated moderation wherein social support mitigates the stigma’s burden impact, providing partial confirmation of Hypothesis 2.

## 4 Discussion

The escalating prevalence of adolescent NSSI has intensified caregiver burden, positioning it as a critical focus in public health research. This study explored the mechanisms linking caregivers’ internalized stigma to caregiver burden, while identifying protective factors. Results confirmed Hypothesis 1, revealing a significant positive association between internalized stigma and caregiver burden. Hypothesis 2 was supported by mediation analyses, demonstrating anxiety’s partial mediation of this relationship. Furthermore, Hypothesis 3 identified social support as a moderating factor, significantly buffering the impact of internalized stigma on anxiety. These findings inform targeted interventions to mitigate caregiver burden through stigma reduction, anxiety management, and social support enhancement in NSSI-affected families.

This study revealed a significant positive association between internalized stigma and caregiver burden, which aligns with previous research (40). The findings highlight that caregivers of adolescents with NSSI often experience significant caregiver guilt, which is frequently interpreted as a reflection of parental inadequacy (41). This cognitive appraisal elicited persistent self-blame cognitions characterized by critical self-evaluations of parental competence (“I failed as a parent”) and subsequent cognitive dissonance regarding pre-existing relational schemas (42). These psychological processes contribute to a complex interplay of emotional distress and relational conflict. Notably, most caregivers reported distressing challenges to their internal working models of parent-child attachment, manifesting as either hypervigilant overprotection or emotional withdrawal patterns (43). Caregivers facing stigma frequently exhibit maladaptive denial of youth mental health issues, resisting professional care due to anticipated social judgment (44). This phenomenon is particularly pronounced in East Asian families, where “face-saving” imperatives may lead to delayed treatment and exacerbate long-term caregiver burden (45). The cultural context of stigma and social norms plays a significant role in shaping caregiver behavior and treatment-seeking patterns. Furthermore, the study highlights the significant financial and emotional burden on families, including reduced working hours, job loss, and substantial financial expenditures on long-term care and therapy (46). These findings underscore the need for comprehensive support systems that address both the psychological and economic dimensions of caregiver burden. To mitigate these challenges, the implementation of multimodal psychoeducational initiatives is recommended. Leveraging digital platforms and social media for evidence-based NSSI education can enhance caregiver awareness and reduce stigma. Peer support programs, which have shown effectiveness in improving caregiver well-being, should be prioritized to address stigma and social isolation (47). Additionally, for socioeconomically disadvantaged families, strategies such as telemedicine and flexible follow-up schedules can reduce financial and logistical barriers to care.

Our findings demonstrate that anxiety mediates 48.94% of the total effect between internalized stigma and caregiver burden among caregivers of adolescents with NSSI, advancing understanding of psychosocial distress mechanisms in this population. This result underscores the complex interplay between psychological distress and caregiving burden, highlighting the role of anxiety as a mediating factor in the relationship between stigma and caregiver burden. Specifically, these results support a qualitative study that self-stigmatizing appraisals (e.g., “I’m to blame for my child’s condition”) not only trigger threat vigilance but also deplete emotional resources, ultimately exacerbating anxiety symptoms that may underlie observed cardiovascular reactivity in stigmatized caregivers (43). The psychological burden of internalized stigma appears to activate a cycle of self-blame and emotional exhaustion, which in turn intensifies anxiety and contributes to physiological stress responses. Moreover, cultural factors are particularly relevant in collectivist societies, where public stigma concerns combined with face-saving behaviors further intensify self-stigma and distress (45). The interplay between cultural norms and psychological distress highlights the need for culturally sensitive interventions that address both individual and societal-level stigma. Notably, chronic anxiety adversely affects both psychological well-being and physical health, contributing to sleep disturbances, immune system dysregulation (48), and ultimately elevating susceptibility to chronic diseases (49). These findings underscore the importance of addressing both psychological and physiological consequences of stigma and anxiety in caregiver populations. Consequently, intervention strategies should integrate psychoeducation to normalize NSSI understanding and policy-level mental health resource allocation (50). From a neurobiological perspective, approaches like MBSR may regulate amygdala hyperactivity in the stigma-anxiety cycle (51), whereas narrative therapy could help reconstruct negative self-perceptions (52), together offering a multifaceted approach to alleviate caregiver burden. These interventions could be particularly effective in addressing the complex interplay between stigma, anxiety, and caregiver burden.

Findings establish social support as a critical moderator attenuating the association between caregivers’ internalized shame and anxiety in the NSSI context. Specifically, social support alleviates caregivers’ shame, thereby indirectly reducing anxiety. This moderating effect occurs primarily in the initial mediation stage, where the shame-anxiety association is weakened by higher perceived social support. Compared to well-supported caregivers, those with lower social support exhibited stronger indirect effects, suggesting that enhancing social support may mitigate anxiety. Consistent with Social Buffering principles, perceived support fortifies psychological defenses to mitigate stressor impacts (53). Within the family systems theory framework, external social support systems mitigate psychosocial stressors’ deleterious impacts on familial homeostasis. Strong social networks bolster collective resilience, improve well-being, and reinforce self-efficacy through positive cognitive reinforcement (42). Notably, parental caregivers, particularly mothers, report higher stigma and burden than sibling caregivers, with female caregivers experiencing greater shame than males (54, 55). Gender differences in emotional processing may underlie this phenomenon, with women typically exhibiting greater emotional sensitivity and irritability vulnerability than men. Additionally, evidence suggests these traits facilitate stronger therapeutic alliances in adolescent care, potentially enhancing empathy-based NSSI management approaches (56). This underscores the need for gender-sensitive interventions, including emotion-regulation training tailored for female caregivers. From a pragmatic standpoint, China’s existing mental healthcare resources remain inadequate, with most concentrated in urbanized eastern coastal regions (57), while community-based mental health services are notably scarce (58). Evidence indicates that caregivers with mental health education report stronger perceived social support, which in turn reduces self-stigmatization. To address these gaps, practical solutions necessitate implementing culturally adapted psychoeducation to enhance mental health literacy, establishing multilevel community networks to strengthen peer-led initiatives, and systematically integrating caregiver support protocols into primary healthcare frameworks to optimize service accessibility through existing infrastructure.

The findings highlight the complex interplay between psychological distress, social support, and caregiver burden in the context of NSSI. The results underscore the importance of addressing internalized stigma, anxiety, and social support in caregiver populations to improve both mental and physical health outcomes. The identification of anxiety as a mediating factor suggests that interventions targeting anxiety may be particularly effective in reducing caregiver burden. Similarly, the moderating role of social support underscores the importance of strengthening social networks and community-based support systems to buffer the negative impacts of stigma and anxiety. From a practical perspective, these findings have significant implications for public health and mental health services. Interventions should prioritize psychoeducation to reduce stigma, promote mental health literacy, and enhance access to mental health resources for families affected by NSSI. Additionally, the integration of digital and community-based support systems could enhance the reach and accessibility of mental health services, particularly in underserved areas. In conclusion, the study provides valuable insights into the mechanisms underlying caregiver burden in the context of NSSI. The findings underscore the importance of addressing psychological, social, and structural factors in developing comprehensive interventions to support caregivers and improve outcomes for adolescents with NSSI.

### 4.1 Limitations

Several limitations warrant explicit acknowledgment. First, the cross-sectional methodology constrains causal interpretation of observed stigma-burden associations, compounded by self-report bias inherent in subjective measures. Future research should adopt longitudinal designs incorporating objective assessments to obtain more accurate data. Second, our sample comprised only caregivers from hospital settings, potentially limiting generalizability. Subsequent research ought to incorporate non-institutionalized caregivers, examining how stigma internalization, anxiety symptomatology, support networks, and caregiver burden dynamically interact.

## 5 Conclusion

This study establishes anxiety as a critical mediator linking internalized stigma to heightened caregiver burden in NSSI-adolescent caregivers, while revealing social support’s moderated role in this mediating pathway. The validated moderated mediation framework necessitates integrated interventions targeting stigma deconstruction, anxiety regulation, and social resource mobilization to alleviate caregiver burden in adolescent NSSI contexts.

## Data Availability

The raw data supporting the conclusions of this article will be made available by the authors, without undue reservation.
